# Evaluating the effectiveness of integrated traditional Chinese and Western treatment based on symptom grading: a study protocol for a multi-center, randomized controlled trial of patients with depressive disorder

**DOI:** 10.3389/fpsyt.2025.1491410

**Published:** 2025-02-12

**Authors:** Jiaxi Mai, Tingwei Zhou, Chen Wang, Junrong Ye, Jiao Chen, Wen Wang, Yuanxin Pan, Yanheng Wei, Lexin Yuan, Hang Yang, Shengwei Wu, Jianxiong Guo, Aixiang Xiao

**Affiliations:** ^1^ Department of Science and Education, Guangzhou First People’s Hospital, Guangzhou, China; ^2^ Department of Geriatric Neuroscience Center, Affiliated Brain Hospital, Guangzhou Medical University, Guangzhou, China; ^3^ College of Nursing, Guangzhou Medical University, Guangzhou, China; ^4^ The Affiliated Brain Hospital, Guangzhou Medical University, Key Laboratory of Neurogenetics and Channelopathies of Guangdong Province and the Ministry of Education of China, Guangzhou Medical University, Guangzhou, China; ^5^ Department of Nursing, Affiliated Brain Hospital, Guangzhou Medical University, Guangzhou, China; ^6^ Department of Traditional Chinese Medicine, Affiliated Brain Hospital, Guangzhou Medical University, Guangzhou, China; ^7^ Department of Chronic Diseases, Affiliated Brain Hospital, Guangzhou Medical University, Guangzhou, China

**Keywords:** depression, integrated traditional Chinese and Western treatment, symptom grading, randomized controlled trials, protocols

## Abstract

**Background:**

Approximately one-third of depressed individuals receive treatment globally. The application rate of traditional Chinese medicine (TCM) for treating depression globally remains relatively low. The proposed study presents a pilot trial to evaluate the effectiveness of interventions at different levels in improving depression status in community populations.

**Methods:**

A randomized controlled trial will be conducted in two communities in Guangdong, China, with a follow-up period of 12 weeks. Participants will be randomly allocated to control or intervention groups. Participants in the control group will be assigned to routine care, while participants in the intervention group will receive TCM intervention measures. The participants in the intervention group will receive integrated traditional Chinese and Western treatment according to the symptom grading of depression severity. Primary outcome measurements include the Patient Health Questionnaire (PHQ-9), the Hamilton Depression Rating Scale (HAMD-17), and the Self-Rating Depression Scale (SDS). Secondary outcome measurements include the Athens Insomnia Scale (AIS), the Epworth Sleepiness Scale (ESS), the Multiple Mental Health Literacy Scale (MMHL), the Short-Form 12 (SF-12), and the Treatment Emergent Symptom Scale (TESS). The data will be collected at baseline (T1), 2 weeks after intervention (T2), 4 weeks after intervention (T3), 8 weeks after intervention (T4), and 12 weeks after intervention (T5).

**Discussion:**

This study will provide an experimental basis for the effectiveness of hierarchical integrated traditional Chinese and Western medicine (ITCWM) in improving the condition of patients with different degrees of depression. At the end of the study, it is expected for the experimental group to have an improvement in depressive symptoms and sleep quality and an enhancement in mental health awareness.

**Clinical trial registration:**

http://www.chictr.org.cn, identifier ChiCTR2300075169.

## Background

Depression is a type of serious mood disorder, and severe depressive symptom would have critical impacts on individuals’ emotional stability and daily activities (1). In 2019, the global incidence of depressive disorder was estimated at 290 million, representing an increase of 59.28% over 1990 (2). Recent research in China reported an 8.29% prevalence of moderate to severe depression in 2021 (3, 4). From 1990 to 2017, the disability-adjusted life years (DALYs) of depression in China had increased by 36.5%, indicating a significant disease burden (3). Research had demonstrated that approximately 1 out of every 10 patients in primary care experience depressive symptoms, and the incidence of depression has also increased in secondary care (5). Mild depression can manifest as anhedonia, sadness, and feelings of worthlessness, whereas severe depression is marked by an ongoing intention to kill oneself (6). Therefore, depression severely limits psychosocial functioning and diminishes life quality (7).

Depression can be treated by using pharmacotherapy (8), psychological intervention (9), or a combination of both (10). Evidence from high-income countries supported the efficacy of antidepressants and psychological interventions for depression. In low- and middle-income countries (LMICs), evidence indicates effective treatment approaches for depression, including low-cost antidepressants, cognitive behavioral therapy, and combined traditional Chinese medicine (TCM) (11, 12). Previous findings showed that integrated traditional Chinese and Western medicine (ITCWM) enhanced treatment outcomes by combining the symptom-targeted approach of Western medicine with the holistic principles of ITCWM. A study by Zhao (13) reported that acupuncture as an add-on therapy to selective serotonin reuptake inhibitors (SSRIs) improved depression symptoms significantly better than SSRIs alone (13). A meta-analysis (14) showed that compared with using Western medicine alone, syndrome differentiation could improve the effect of treatment [WMD = −2.39, CI (−2.96, −1.83), *Z* = 8.29, *p* < 0.001] (14).

Clinically, SSRIs are a commonly used antidepressant, and studies showed that patients would experience side effects such as weight gain (15), sexual dysfunction (16), and gastrointestinal side effects (such as nausea, vomiting, and constipation) (17) after using SSRIs. Side effects might have an impact on treatment adherence (18), and a survey reported that up to 43% of patients with major depressive disorder drop out of antidepressant treatment due to side effects (19). TCM is widely applied in Asia, particularly in low- and middle-income and developing countries (20); 13.6% of individuals with mental disorders would seek help from TCM, and our earlier investigation indicated that leading psychiatrists supported the use of TCM to alleviate depressive symptoms and side effects caused by antidepressants and that most of them had prescribed TCM (75%) and had recommended TCM to junior psychiatrists (65%) (21). Therefore, an integrated traditional Chinese and Western approach is expected to provide a new direction of managing depression by maintaining medication, increasing treatment adherence, and reducing side effects, concurrently. Although Western medicine has accumulated experience in treating depression, TCM has made great progress in treating depression. By reviewing the literature, we found that comprehensive health interventions based on TCM aimed at educating patients and changing their lifestyle, including exercise (e.g., Tai Chi and Qigong), diet therapy, sleep–wake schedule, psychological management based on TCM theory, and meridian and collateral management (14, 22). Treating depression by TCM has significant effects with less toxic side effects or relapse (23). Studies had demonstrated that TCM, particularly herbal medicine and acupuncture, had significant effects in alleviating depressive symptoms. A meta-analysis by Ching et al. (24) reported that TCM-based treatments were effective in reducing depression severity, with fewer side effects compared to using Western medicine alone (MD: −8.73, 95% CI: −16.64, −0.79) (24). Moreover, clinical evidence indicated that the combined therapy of ginger-isolated moxibustion and escitalopram achieved a remarkably effective rate and reduced relapse rates (25). Integrated traditional Chinese and Western treatment significantly optimizes the treatment effect and improves the prognosis, especially in LMICs. Additionally, our preliminary investigation discovered that leading psychiatrists advocate using TCM (23). Thus, exploring the combination of traditional Chinese and Western medicine in treating depression to leverage the advantages of TCM is necessary.

This policy-supported study aims to explore a new depression prevention and treatment mode to promote patient recovery and reduce depression recurrence. This mode adds TCM elements to improve the therapeutic effect. Simultaneously, according to the different depression levels in patients, multi-level ITCM interventions are expected to improve depression. We recruited a psychiatric treatment team to consult the feasibility and acceptability of the proposed program according to the Integrative Chinese and Western Medicine guidelines issued by China Association of Chinese Medicine (https://www.cacm.org.cn/) (26), the largest Chinese medicine academic association supervised by the National TCM Administration. Finally, experts had reached the consensus of a proposed model after discussion.

We propose a multi-center study to evaluate the effectiveness of hierarchical integrated traditional Chinese and Western medicine (ITCWM) care in depression patients using a randomized controlled trial design.

## Objectives

Based on empirical findings, a program has been developed to improve the depressive state of patients with different degrees of depression using ITCWM. This study intends to evaluate the effectiveness of integrated traditional Chinese and Western treatment in improving depression in patients with different depression levels.

## Methods

### Trial design

The proposed program is a multi-center, exploratory, double-blind, parallel randomized controlled trial (RCT) with a follow-up period of 12 weeks. All stages will be conducted by SPIRIT reporting guidelines (27). Treatments administered will be allocated randomly according to a 1:1 ratio using an online random-number generator of statistic software by a research assistant who is not involved in assessment and intervention. The block size for randomization will be 4 to ensure balanced group allocation. To maintain allocation concealment and reduce bias, the randomization sequence will be generated by an independent research assistant who is not involved in participant recruitment or data collection. Opaque and sealed envelopes will be prepared according to the randomization sequence and provided to the patients by the principal investigator or research assistants in the community. Then, research assistants would be responsible for monitoring participants’ engagement and adherence to the intervention protocol.

### Study settings and participants

This trial will be conducted in two community health service centers with similar administrative conditions in Liwan District, Guangzhou. Simultaneously, referral support will be accepted from the Affiliated Brain Hospital of Guangzhou Medical University. Ethical approval was obtained from the Institutional Review Committee of Guangzhou Medical University. This study will be conducted with the consent of hospital executives. Before allocation, informed consent will be obtained from all participants.

### Eligibility criteria

#### Inclusion criteria

- Comply with the diagnostic criteria for depressive episodes in the International Classification and Diagnostic Standards of Diseases (28).
- Aged 18 and 70 years.
- The score of PHQ-9 is 5 or above.
- Participants are informed and signed the informed consent form voluntarily.

#### Exclusion criteria

- Pregnant women and lactating women.
- Severe cognitive impairment. The cognitive level is evaluated using the Minimum Mental State Examination (MMSE). The MMSE is a paper‐based test with a maximum score of 30, with lower scores indicating more severe cognitive problems. The cutoff point for normal cognitive function is typically set at 24 (29).
- Those with severe heart, liver, kidney disease, and other systemic diseases requiring treatment.

### Trial status

Client recruitment started on 1 October 2023 and ended in July 2024.

### Interventions

**Figure 1:**
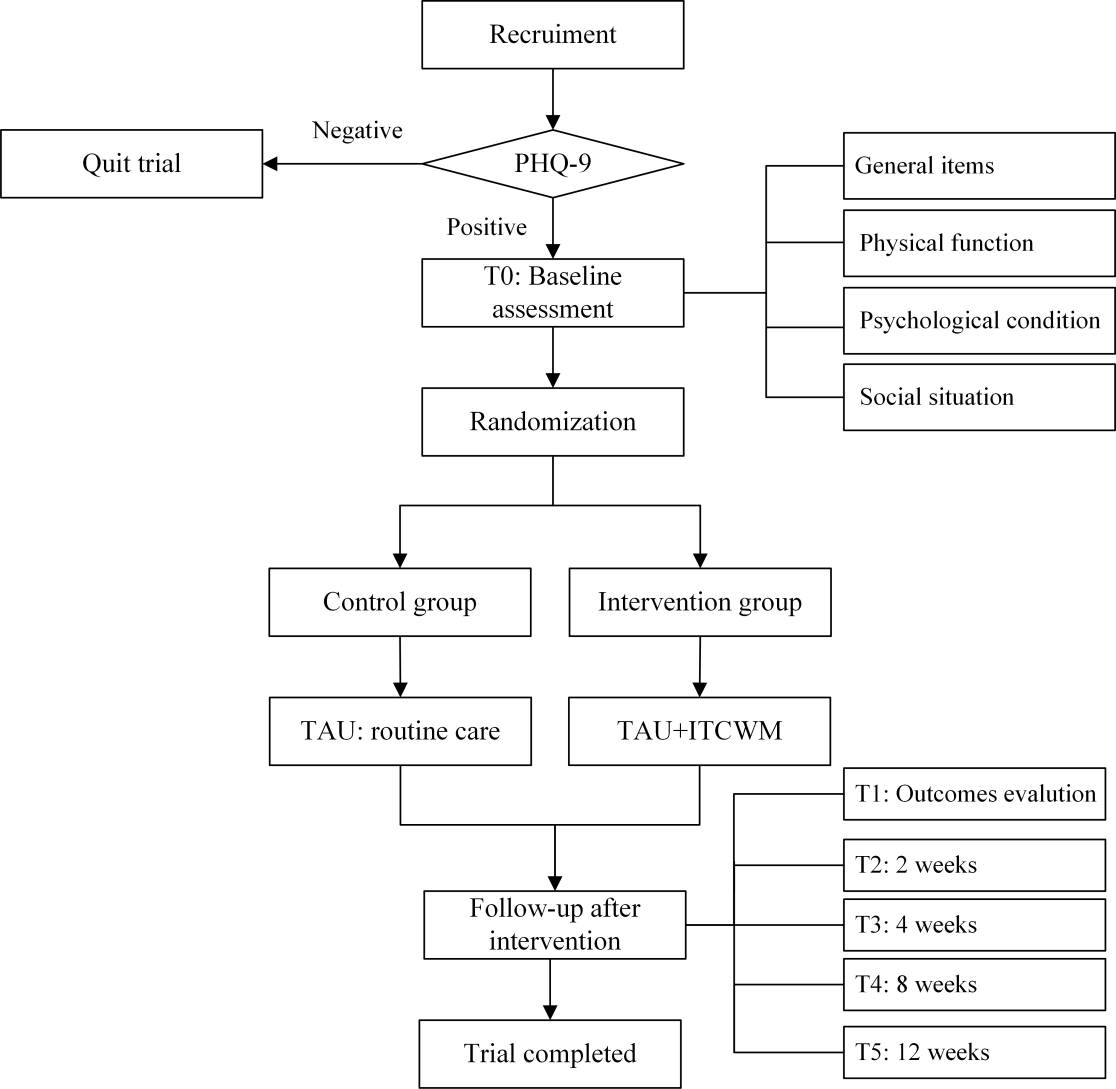
The study’s flowchart.

**Figure 2:**
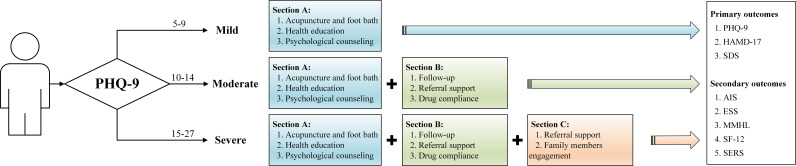
Integrated traditional Chinese and Western treatment graded intervention flowchart.

Participants will be randomly assigned to an intervention or a control group. The flowchart of this study is presented in **Figure 1**. The control group will receive routine care, while the intervention group will receive special ITCM intervention measures for 12 weeks. The participants included in the intervention group will undergo intervention according to **Figure 2**.

According to PHQ-9, participants will be divided into three levels: mild, moderate, and severe, and will receive corresponding interventions. The intervention strategies are dynamically adjusted based on the evaluation of time nodes. **Table 1** presents a detailed study’s graded intervention.

**Table 1:** The details of graded interventions applied to the groups.

	Group allocation		Intervention group			Control group
Intervention	PHQ-9 (score)		Mild (5–9)	Moderate (10–14)	Severe (15–27)	Positive (5–27)
	Section A	1. Acupuncture and foot bath	√	√	√	1. Health education 2. Psychological counseling
		2. Health education				
		3. Psychological counseling				
	Section B	1. Follow-up		√	√	
		2. Referral support				
		3. Drug compliance				
	Section C	1. Referral support			√	
		2. Family members engagement				
Time	2, 4, 8, and 12 weeks					

#### Section A

1. Therapeutic acupuncture

Acupuncture protocol was according to the Standards for Reporting Interventions in Controlled Trials of Acupuncture (STRICTA) (30). A total of three licensed TCM practitioners with ≥5 years’ work experience are responsible for selecting the acupoints. All TCM practitioners will receive consistency training according to study protocols. The selection of acupoints will be based on the established TCM principles and tailored to each participant’s specific presentation of depression.

First, the TCM practitioner would conduct a thorough evaluation of each participant, including their symptoms, medical history, and overall health status, to determine the most appropriate acupoints. Furthermore, based on this assessment, acupoints will be selected from well-established protocols for depression treatment in TCM. The primary acupoints include Baihui (GV 20), Qihai (CV 6), Neiguan (PC 6), Shenmen (HT 7), Guanyuan (CV 4), Taichong (LR 3), Sanyinjiao (SP 6), and Zusanli (ST 36). When performing manual acupuncture, the needle should be inserted slowly and retained for 30 min after getting qi. The needle type is a sterile disposable stainless-steel needle size of 0.35 × 25 mm. Acupuncture courses are scheduled three times a week for 30 min each (31).

2. TCM foot bath

The participants will sit on a chair with the water temperature at 40℃. The water depth is 10 cm above the ankle.

3. Health education

Health education information can be regularly released via online psychological forums and WeChat official accounts. Simultaneously, promotional brochures will be distributed to help participants understand depression-related knowledge. Health education focuses primarily on depression, including etiology, pathogenesis, clinical manifestations, prognosis, treatment, and rehabilitation guidance. The focus is on the effects of antidepressants, adverse reactions, and management of antidepressants.

4. Psychological counseling

Participants initiated in-person psychological counseling with trained nurses. This approach is based on the assumption that communicating with a non-judgmental and empathic professional enables individuals to take a more positive view of themselves and their lives (32).

#### Section B

1. Follow-up

All participants will be subjected to follow-up at 2, 4, 8, and 12 weeks. The follow-up includes physical, psychological, and social conditions. The medication status, referral status, recurrence status, and economic burden will be collected using self-made scales.

2. Referral support

For participants with mild (PHQ-9 score 5–9) and moderate depression (PHQ-9 score 10–14), the experimental community will complete evaluation, treatment, and follow-up according to the intervention plan in the community. If the condition worsens or the treatment conditions cannot be met, the participants can be transferred to a superior hospital. Moreover, the participants will be transferred to primary healthcare for continued rehabilitation after treatment and recovery at the superior hospital.

3. Basic medication

SSRIs, for example, sertraline and fluoxetine, will be used to alleviate depressive symptoms (33). When insomnia occurs, combining zolpidem, zopiclone, and short-acting benzodiazepine sedatives could improve sleep. Additionally, when it comes to medication for treating physical diseases, maintaining a consistent type and dosage of medication can help to reduce confounding. To ensure consistency in medication use, all participants receiving SSRIs would follow a uniform prescribing protocol, including dosage adjustments and monitoring by the same team of qualified clinicians. Additionally, participants’ medication regimens would be closely tracked, and any changes would be documented and accounted for in the statistical analysis to isolate the effects of the integrated traditional Chinese and Western intervention. During the study, antidepressant medications would be applied based on the guideline for primary healthcare service of major depressive disorder (34) introduced by the Chinese Medical Association.

#### Section C

1. Referral support

Participants with severe depression (PHQ-9 score 15–27) exhibit suicidal or homicidal thoughts or intentions after completing assessments regarding mental, suicide risk, and violence. Once a participant is evaluated as experiencing severe depression (PHQ-9 ≥15) and exhibited suicidal or homicidal thoughts, they would be referred to specific psychiatric hospitals for close monitoring and treatment. In addition, for participants with severe depression but did not exhibit high-risk behaviors, our research team would provide ongoing monitoring and support throughout the study period.

2. Family engagement

2.1 Communication with family: Participants will be encouraged to communicate their feelings and experiences with their families. Encouraging family members to understand patients’ conditions, involving them in treatment, and urging them to provide support can help the participants.

2.2 A brochure is developed for the family-enhanced depression intervention. The manualized intervention is delivered by nurses in primary care and consisted of up to 12 sessions delivered in the primary care clinic over 4 weeks. A participant’s family member would be encouraged to participate in all sessions of the intervention. The intervention included psychoeducation regarding depression and treatment, self-management support (medication adherence and questions with medications), behavioral activation, guidance on primary care physician visits, and relapse and prevention planning. A family member can participate in treatment sessions as part of the intervention to support one or more of these aspects of depression care (35).

If a participant misses any treatment, the research assistants will contact them.

### Routine nursing care during intervention

Participants and their caregivers received routine care and health education during the intervention and follow-up period. Treatments include observation, pharmacological therapies, and follow-up at the same time as the intervention group.

## Outcomes

### Primary outcomes

The PHQ-9 would be used to evaluate symptoms of depression during the past 2 weeks. The total score ranges from 0 to 27 with five categories of severity: minimal (0–4), mild (5–9), moderate (10–14), moderately severe (15–19), and severe (20–27) (36, 37).

The HAMD-17 (38) is used to assess the severity of depressive symptoms in participants. This scale has good reliability and validity and is widely used to assess the severity of depressive symptoms. The depression severity is classified according to the total score of HAMD-17: 0–7 points for no depressive symptoms, 8–17 points for mild depressive symptoms, 18–24 points for moderate depressive symptoms, and >24 points for severe depressive symptoms.

The SDS (39) has 20 items designed on the was basic of diagnostic criteria for depression. Participants rate each item about how they have felt during the past several days. The raw sum score of the SDS ranges from 20 to 80, but results are usually presented as the SDS Index, obtained by converting the raw score to a 100-point scale.

### Secondary outcomes

The AIS is a self-assessment psychological measurement instrument aiming to quantify sleep difficulties according to the ICD-10 standard. The scale consisted of eight items. The total score ranges from 0 to 21. The questions are scored from 0 to 3 according to the Likert scale, and a total score ≥6 indicates insomnia. High scores indicate severe insomnia symptoms (40).

The ESS (41) would be used to assess daytime sleepiness. This questionnaire consists of eight self-rated items, each scored from 0 to 3, which measure participants’ habitual “likelihood of dozing or falling asleep” in common situations of daily living. The final score was the sum of individual items (scores 0–24). Values > 10 are considered excessive daytime sleepiness, whereas values > 15 are considered severe sleepiness.

The MMHL, developed by Jung et al. (42) in 2012, would be used to measure adults’ mental health literacy level. Zhijun et al. (43) conducted the 22-item Chinese translation, including the following three dimensions: mental health knowledge, beliefs, and resources. The total score ranges from 0 to 22. Higher scores suggest a higher level of mental health literacy.

The SF-12 is a 12-item instrument designed to measure general health-related quality of life (HRQoL) (44). Answers provide two summary scores, the mental component scale (MCS) and the physical component scale (PCS), reflecting the individual’s perceived mental and physical health. Scores range from 0 to 100 for each component scale. A score of 50 or more indicates a positive perception of health, whereas a score below 50 indicates a negative perception.

The TESS (45) was developed by the National Institute of Mental Health (NIMH) in 1973. TESS includes common adverse symptoms and signs and numerous laboratory test results. This scale has the advantage of allowing the symptoms of each system to be fully identified.

### Sample size

This RCT calculates sample size by comparing the mean of two samples. The HAMD-17 score is used as the outcome indicator for observation. According to a previous study (46), the mean of HAMD-17 was 13.3 in the control group, with a standard deviation of 3.6, while the mean of HAMD-17 was 16.5 in the intervention group, with a standard deviation of 4.6. PASS 15.0 version was used to estimate the target sample size, 1 − β = 0.9, α = 0.05. Lost and refused visits were considered, and a 20% dropout rate was calculated. Therefore, the target sample size for each group will be 47 participants. A total of 94 participants will be targeted.

### Recruitment

Participants who meet the screening requirements in this study will be invited. Every participant has finished the initial step of evaluation, which is a regular process throughout the early phases of the investigation. The participants will be provided with all the necessary information about the study. The research team will inform patients of the research purpose and process and fully respect the rights of the participants.

### Allocation

The research sites for the experiment are two community health service centers with similar administrative conditions in Liwan District, Guangzhou. Consecutive eligible participants will be collected to reduce selection bias until the number of cases assigned is completed.

### Blinding

This is a double-blind study. The concealment and blinding of the assignment will be assured since the assistants performing the assessment will be unaware of the subject’s group assignment. This study’s results will be evaluated by trained research assistants. Participants will not be informed of the pattern of research allocation. During the intervention, the veil of blinding will be lifted if severe complications and adverse events occur. During the trial, both participants and research assistants conducting psychological assessment would not be disclosed about group allocation. Furthermore, research assistants conducting interventions would be required to only implement psychological counseling while not disclosing group allocation.

### Data collection and management

The impacts of traditional Chinese and Western medicine intervention will be collected at baseline (T1), 2 weeks after intervention (T2), 4 weeks after intervention (T3), 8 weeks after intervention (T4), and 12 weeks after intervention (T5) to dynamically adjust intervention measures based on intervention effectiveness. Other outcomes, including adverse events, attendance, and attrition rates, will be continuously collected weekly from baseline. Research assistants will be trained to ensure consistent results. To ensure an adequate follow-up rate, we will maintain regular contact with patients after giving informed consent (every 2 weeks). After the measurement, data will be double-checked for consistency, accuracy, and integrity. Raw data will be anonymized and stored on a secured computer database. According to the regulation governing research activities, data will be monitored by Department of Research Project Administration of GZ. Research data could only be accessed by authorized members of the trial team. After obtaining permission from the project investigator (AX), anonymized trial data would be shared to enable further prospective meta-analyses.

### Statistical methods

SPSS 25.0 statistical analysis software will be used for statistical analysis. The continuous variable will be expressed in mean ± standard deviation (*χ* ± SD) and frequency (%). The SNK-q test will be used to compare the two groups, and the analysis of variance will be used to compare the measurement data at multiple time points. All *p*-values are two-sided and reported as statistically significant based on a significance level 0.05. We shall study protocol non-adherence during the process analysis and will impute missing data by multiple imputation.

### Monitoring

All adverse events spontaneously reported by participants or observed by the research team will be recorded. A research assistant will be assigned to conduct a biweekly review of the study progress and any adverse events of this study.

### Ethical issues

The trial will comply with the Helsinki Declaration of Human Rights principles. This research has obtained ethical approval from the Ethics Committee of the Affiliated Brain Hospital of Guangzhou Medical University (approval number: 2023062).

### Modification and inquiry

Any modification or inquiry regarding the study will be communicated to the Ethic Committee of the Affiliated Brain Hospital of Guangzhou Medical University.

### Consent

Written informed consent forms will be collected from participants before the allocation by research assistants. Participants will be informed that they can accept or refuse participation in the study. On the consent form, participants will be asked if they agree to use their data if they withdraw from the trial. Participants must also obtain permission from the research team to share relevant data with other reasonably purposeful research teams and regulatory agencies. If a participant’s condition suddenly deteriorates or severe adverse reactions occur during the experiment, the experiment will be immediately halted, and researchers will administer active medical measures. If participants have a high risk of suicide, our researchers will also provide preventive measures. Participants will be informed that they can accept or refuse to participate in the study.

### Confidentiality

The participant’s personal data, including name, address, and phone number, included in the consent form and case report form, will be anonymized using the study ID number and stored in a secured database. The research data will be monitored by the Research Project Management Department of Guangzhou Medical University, and only authorized members of the experimental team can access it.

### Post−trial care

If the study demonstrates no negative effects, participants in the control groups will be invited to participate in the integrated traditional Chinese and Western treatment based on the symptom grading program after the study time frame.

### Access to data

The datasets will be available from the corresponding author upon reasonable request.

### Dissemination

This study’s results will be disseminated through peer-reviewed publications and conference presentations. After completing the trial, the results will only be used for the proposal. This plan can be publicly accessed via the above clinical trial registration platform. The research results will also be presented in peer-reviewed scientific journals and published at academic conferences.

### Study quality control

The tools utilized in this study are subjected to reliability and efficiency tests. All research assistants will receive consistency training to conduct appropriate assessments and review questionnaires to improve homogeneity. Additionally, the researchers are trained on how to assess and implement the interventions and are allowed to check their quality each month.

## Discussion

This protocol proposes an exploratory RCT to evaluate the effectiveness of ITCWM care in improving the depressive status of patients with different degrees of depression in Guangdong, China. This trial course will comprise a 12-week intervention and a 12-week follow-up period.
